# Supplementary material for: Incidence and etiology of chronic pulmonary infections in patients with idiopathic pulmonary fibrosis
Source: PLoS One. 2020 Apr 3;15(4):e0230746. doi: 10.1371/journal.pone.0230746 (PMC7122739; doi:10.1371/journal.pone.0230746)
Supplement: Attachment — Submitted filename: Response to Reviewers.doc [file pone.0230746.s001.doc]

Title: “Incidence and etiology of chronic pulmonary infections in patients with idiopathic pulmonary fibrosis” Manuscript ID PONE-D-19-31648 by Odashima K et al.

Dear Dr. de Jesus Perez and Reviewers 1 and 2:

Thank you very much for giving us the opportunity to revise our manuscript. We are grateful to you all for the helpful comments concerning the previous revised version of our manuscript, and we have made every effort to prepare this version of our paper accordingly. The main document and tables were revised. Revised sentences in the manuscript are written in red color. Our point-by-point replies to the editor’s and reviewers’ comments follow below.

We believe that the quality of the revised manuscript is now improved according to your invaluable comments, and we hope that you will now find this revised version of our paper suitable for publication in PLOS ONE.

***Journal Requirements to Author:***

1. ***Please ensure that your manuscript meets PLOS ONE's style requirements, including those for file naming.***

We changed our manuscript to meets PLOS ONE’s style requirements.

***2.*** ***Please specify in your ethics statement whether participant consent was written or verbal. If verbal, please also specify: 1) whether the ethics committee approved the verbal consent procedure, 2) why written consent could not be obtained, and 3) how verbal consent was recorded.***

The ethics committee approved the verbal consent procedure. We used an opt-out method and disclosed it to the hospitalized patients. No patient refused to participate. We added the following to the text:

Page 6, line 87

The ethics committee of the same hospital approved the verbal consent procedure. We used an opt-out method and disclosed this to the hospitalized patients. No patient refused to participate.

3. ***We noticed you have some minor occurrence of overlapping text with previous publications, which needs to be addressed. In your revision ensure you cite all your sources (including your own works), and quote or rephrase any duplicated text outside the methods section. Further consideration is dependent on these concerns being addressed.***

Because the patient groups in the first paper are from the same institution, they are considered to be partially overlapping, but they were not identified because the purpose of that study was completely different. We revised the wording of the duplicate text.

***Review Comments to the Author***

***Reviewer #1:***

***“Five- and ten-year mortality rates of patients with and without CPI development were 43.4% and 33.0%, and 68.9% and 72.8%, respectively.” In this statement in mortality section, are they trying to say that mortality is higher in those without infections? Although there was no difference in the KM curve. Please explain***

Thank you for your valuable comments and question. There were no significant differences in mortality between the two groups. We are trying to say that the presence or absence of CPI did not affect mortality. We added the following to the text:

Page 18, line 186

Thus, the presence or absence of CPI did not affect mortality.

***The authors found KL 6 levels to be associated with survival in those with CPI. How do they explain this finding when KL6 level was elevated with diagnosis of IPF.***

Thank you for your question. Increased serum KL-6 in interstitial lung disease reflects impairment and the production of type II alveolar epithelial cells and is strongly correlated with fibrotic lesions.

(Am Rev Respir Dis. 1993;148:637-642)

(Respir Med. 2010 Jan;104(1):127-33)

In addition, serum KL-6 levels are known to be high in infections such as pulmonary tuberculosis, and destruction of lung tissue due to infection is thought to be one of the causes. Therefore, the prognosis of the lung infection cases with structural changes (structural modification) in the lungs may have been worse.

(Tubercle lung Dis 1995 ; 76 : 230-233.)

From the above two points, in interstitial pneumonia with CPI, KL-6 is elevated, and we consider that progression of lung lesions affects survival.

We added the following to the text:

Page 24, line 293

Increased serum KL-6 in IPF reflects impairment and the production of type II alveolar epithelial cells and is strongly correlated with fibrotic lesions. In addition, serum KL-6 levels are known to be high in infections such as pulmonary tuberculosis, and destruction of lung tissue due to infection is thought to be one of the factors. Therefore, the prognosis of the patients with lung infection and structural changes (structural modification) in the lungs may have been worse. From the above two points, in IPF with CPI, KL-6 is elevated, and we consider that progression of these lung lesions can affect survival.

***I would recommend to add a separate table on KL6 data findings. Showing how its different in each group and the trend in those who had CPI. This is an interesting finding of this paper as KL6 is not routinely used for treatment or evaluation of IPF world wide.***

Since a univariate analysis on KL-6 values was already performed in the first manuscript, we thought that it would be of little significance to create another table for KL-6. Therefore, the revised manuscript does not include a new table. However, we appreciate your valuable suggestion regarding KL-6 values.

***Regarding diagnosis of IPF, the authors have mentioned using ATS criteria. By which I am assuming all these patients had HRCT and a multidisciplinary evaluation. If yes, it would be informative to know how many of these were definite IPF or probable or possible and then showing the prevalence of these infection based on the type of IPF.***

Thank you for your comments. All patients had HRCT and a multidisciplinary evaluation. They were diagnosed as having definite IPF.

***Reviewer #2:***

***The authors institution is a tertiary referral care for non-tuberculosis respiratory diseases and tuberculosis. Given that, it is probably reasonable to resume that it is also a referral center for complex patients with nontuberculous mycobacteria and other chronic infections. Given that older patients may be predisposed to these infections, and that the same population group is at a high risk for idiopathic pulmonary fibrosis as well, unless we can factor in the denominator, in addition to the expected baseline rate of idiopathic pulmonary fibrosis, nontuberculous mycobacteria, tuberculosis and other infections, one can question whether the association between idiopathic pulmonary fibrosis and chronic infections is spurious, and is secondary to a referral bias. For example, if the expected prevalence of idiopathic pulmonary fibrosis in a population is 10% and the expected prevalence of chronic infections is 5%, then just by chance alone, 0.5% of patients will have both the diseases, and if these patients are somehow referred to a single institution, simply analyzing these patients with both diseases can give a false impression that there is an association when there actually is not. This could be a difficult task to accomplish but the authors should try to address this in the manuscript.***

Thank you for your valuable feedback. In line 246, as mentioned in the discussion, because our hospital has 51 beds for TB and TB patients tend to be referred to our hospital, it would be reasonable for *M. tuberculosis* to be the most frequent etiology of CPI at IPF diagnosis. There may be referral bias.
However, no patient was referred for suspected NTM although referral bias from visits for cough or sputum workup cannot be completely ruled out. As described from line 253, regarding NTM in the present study, the incidence density of NTM lung disease and MAC lung disease was 9.71 and 8.04 cases per 1000 person-years, respectively. Thus, the incidence of NTM in patients with IPF would be higher than that in the general population.

There might be a bias toward TB, but the results of NTM suggest that patients with IPF have a greater risk of developing chronic infections than does the general population.

We added the following to the text:

Page 25, line 318

Finally, our institution is a tertiary referral care for non-tuberculosis respiratory diseases and tuberculosis. Because our hospital has 51 beds for TB and TB patients tend to be referred to our hospital, it would be reasonable for TB to be the most frequent etiology of CPI at IPF diagnosis. Thus, there may be referral bias. However, no patient was referred for suspected NTM although referral bias from visits for cough or sputum workup cannot be completely ruled out. In the present study, the incidence density of NTM and MAC was 9.71 and 8.04 cases per 1000 person-years, respectively. Thus, the incidence of NTM in patients with IPF would be higher than that in the general population. There might be a bias toward TB, but the results of NTM suggest that patients with IPF have a greater risk of developing chronic infections than does the general population.

***The authors have divided the groups based on when IPF and CPI respectively were diagnosed in relationship to each other. Table 1 mentions steroids and immunosuppressants in these patients without alluding to the temporal relationship between the infection and immunosuppression. It probably makes more sense to divide the groups based on whether these patients had no risk factors, or had risk factors such as being immunosuppressed, or receiving corticosteroids before a diagnosis of CPI was made. These subgroups can be further divided based on the whether the IPF was diagnosed first, CPI, or at the same time.***

Thank you for your valuable comments. Your proposal may be valid, but this study sought to determine whether the presence of CPI at the time of IPF diagnosis could affect the subsequent course of IPF. As a result, we did not split the groups in the way suggested by the reviewer.

***Group C includes patients who had chronic infections at the time of diagnosis but none at the time of follow-up. Is this because these infections were treated? Or do they represent false positives?***

No CPI at the time of follow-up means that no new CPI occurred in the subsequent course of IPF.

***Table 3. Multivariate Cox progression final model. ESR. It is unclear why there are multiple groups for this. Perhaps best to ignore those with unknown ESR and divide the rest into 2 groups only, less than 20, and more or equal to 20.***

Thank you for your comments. As you pointed out, there was a trend, but there was no significant difference. However, there is a possibility that a significant difference may occur at a higher ESR value, so we divided the classifications to include ≥ 40 mm/h (Some readers might think so, so we included a group with higher ESR value). We did not make any changes in this regard.

***Figure3. If the difference between these 2 groups is not significant, it is unclear how this figure adds to the manuscript.***

Thank you for your valuable comments. There was no significant difference in mortality between the two groups. However, we thought it was important to emphasize that the presence or absence of CPI did not affect mortality. For this reason, we did not make any changes in this regard.

***Figure 4. Given the numerous factors affecting mortality in this group, this is likely not needed.***

Thank you for your comment. Your point is reasonable, but we would like to clarify the mortality rate of IPF after the onset of CPI in terms of whether there is any significant divergence from previous IPF mortality. We did not make any changes in this regard.

***Table 4. To patients with microscopic polyangiitis were included in the group. Usual interstitial pneumonia pattern has been well described in patients with microscopic polyangiitis, and can in fact precede the diagnosis of microscopic polyangiitis. This patients do not have IPF and should not be included in the group.***

Thank you for your comments. In a group of patients initially diagnosed as having IPF, some of them may meet the criteria for MPA later. Therefore, we believe that MPA is a complication of IPF and can be included in the causes of death in patients with IPF.

(BMJ Open Respir Res. 2015 Jan 9;2(1))

***Did all the patients with the organism isolates meet relevant clinical criteria for infection?***

Yes, they did. All of the patients with the organism isolates met the relevant clinical criteria for infection. If patients fulfilled the diagnostic criteria for mycobacterial tuberculous lung diseases of the 2015 Japanese Society for Tuberculosis of Japanese guidelines, TB was diagnosed when *Mycobacterium tuberculosis* was cultured from respiratory specimens. If patients fulfilled the diagnostic criteria for nontuberculous mycobacterial lung diseases of the 2007 American Thoracic Society/Infectious Disease Society of America guidelines, they were diagnosed as having NTM. According to the 2014 Committee guidelines of deep mycosis, CPA was diagnosed if patients fulfilled these diagnostic criteria: 1) compatible symptoms continuing >1 month; 2) new consolidation or mass with/without cavity formation, or new consolidation with preexisting cavity expansion; and 3) positive isolation of *Aspergillus* species from respiratory samples or positive serum antibodies to *Aspergillus.* According to a previous paper, Nocardiosis was diagnosed when *Nocardia* species was cultured from respiratory specimens along with compatible symptoms and new consolidation.

(The Japanese Society for Tuberculosis. 2015 Guideline for Tuberculosis, 3rd Edition. Tokyo: Nankodo; 2015.)

(Committee guidelines of deep mycosis. 2014 Guidelines for the diagnosis and treatment of deep mycosis. Tokyo: Kyowa Planning; 2014.)

(Respir Investig. 2014 May;52(3):160-6)

We also added the following to the methods in the text:

Page 7, line 97

If patients fulfilled the diagnostic criteria for mycobacterial tuberculous lung diseases of the 2015 Japanese Society for Tuberculosis of Japanese guidelines,…

Page 7, line 102

According to the 2014 Committee guidelines of deep mycosis,…

Page 7, line 108

Referring to a previous paper,…

***The authors should speculate on the reason for CPI in patients with IPF and hypothesize on possible mechanisms.***

Thank you for your suggestion. Patients with altered lung structure have reduced local immunity and are more susceptible to infection. CPI is likely to be secondary to the degree of structural destruction underlying the lung parenchyma found in IPF, but further studies are needed on the influence of pathogenic mechanisms.

(Committee guidelines of deep mycosis. 2014 Guidelines for the diagnosis and treatment of deep mycosis. Tokyo: Kyowa Planning; 2014.)

We added the following to the text:

Page 23, line 284

As noted above, patients with altered lung structure have reduced local immunity and are more susceptible to infection. CPI is likely to be secondary to the degree of structural destruction underlying the lung parenchyma found in IPF.

Thank you again for your kind comments and advice.

Kyuto Odashima

Department of Respiratory Medicine, Saitama Cardiovascular and Respiratory Center
